# Diabetic Foot Ulcers in Pakistan: A Silent Epidemic?—A Meta‐Analysis of Observational Studies

**DOI:** 10.1002/jfa2.70117

**Published:** 2025-12-25

**Authors:** Reza Ghanei Gheshlagh, Mohammad Esmaeelzadeh, Kainat Asmat, Simin Sharafi

**Affiliations:** ^1^ Nursing Department, Faculty of Health Sciences Biruni University Istanbul Turkey; ^2^ Social Determinants of Health Research Center Research Institute for Health Development Kurdistan University of Medical Sciences Sanandaj Iran; ^3^ Student Research Committee Birjand University of Medical Sciences Birjand Iran; ^4^ Shifa Tameer‐e‐Millat University (STMU) Islamabad Pakistan; ^5^ Department of Nursing School of Nursing and Midwifery Birjand University of Medical Sciences Birjand Pakistan

**Keywords:** diabetes, diabetic foot ulcer, meta‐analysis, Pakistan

## Abstract

**Background:**

Diabetic foot ulcers (DFUs) are a major complication of diabetes, leading to severe morbidity and high healthcare costs. Despite numerous studies on the prevalence of DFUs in Pakistan, the findings vary widely, making it difficult to obtain a clear estimate. To address this gap, we conducted a meta‐analysis to determine the overall prevalence of DFUs among diabetic patients in Pakistan, providing a more comprehensive understanding of the burden of this condition.

**Methods:**

A systematic search was conducted in PakMediNet, PubMed, Scopus, Web of Science, and Google Scholar without language or time restrictions. Two independent reviewers screened studies, extracted data, and assessed quality. Heterogeneity was evaluated using Cochran's Q and I^2^. Subgroup analysis, meta‐regression, and sensitivity analysis were performed. Publication bias was assessed via a funnel plot and Egger's test. The meta‐analysis was conducted using a random‐effects model in Stata 17.

**Results:**

In this meta‐analysis, 16 studies with a total of 15,333 participants were analyzed. The pooled prevalence of DFUs was estimated at 18%, with a prevalence of 19.4% in Punjab and 16.6% in other regions. The prevalence was reported as 20.8% in men and 14.9% in women. Sensitivity analysis indicated that the results were highly stable. Meta‐regression revealed a significant increasing trend in the prevalence of DFUs from 1999 to 2022. Although statistical tests confirmed the presence of publication bias, the trim‐and‐fill method suggested that its impact was negligible.

**Conclusion:**

This meta‐analysis provides a comprehensive estimate of the prevalence of DFUs, highlighting regional and gender differences. The findings show a significant increasing trend over time, emphasizing the growing burden of this condition. These results highlight the need for targeted prevention strategies and improved management of DFUs.

## 1 Introduction

Diabetes is one of the greatest health challenges of the 21st century, affecting millions of people worldwide [1]. This disease is characterized by elevated blood sugar levels due to defects in insulin production or function and is associated with various complications [2]. Diabetes is recognized as a global concern not only because of its acute and chronic complications but also due to the economic and social burden it imposes on healthcare systems and societies [3]. In 2022, around 828 million adults aged 18 and older had diabetes, an increase of 630 million compared to 1990. This rising trend is particularly alarming in low‐ and middle‐income countries, including Pakistan [4].

Diabetes leads to numerous serious complications, including cardiovascular diseases, nephropathy (kidney damage), retinopathy (damage to the retina), neuropathy (nerve damage), and diabetic foot ulcers (DFUs) [5]. DFU are among the most severe complications of diabetes, potentially resulting in infections, amputations, and even death. These ulcers develop due to a combination of peripheral neuropathy, reduced blood flow to the feet (ischemia), and impaired wound healing [6]. DFU not only significantly impact patients' quality of life but also impose a substantial financial burden on healthcare systems. Studies indicate that the direct and indirect costs associated with DFUs amount to billions of dollars worldwide [7]. These costs include medical expenses, prolonged hospitalizations, surgeries, and postamputation care [8].

In countries, such as Pakistan, where the healthcare system faces financial and structural constraints, this issue is even more critical. Pakistan, with a population of over 240 million, is one of the countries facing a growing prevalence of diabetes. According to recent reports, more than 33 million people in the country have diabetes and this number is increasing due to lifestyle changes, unhealthy diets, and physical inactivity [9]. In such circumstances, DFU are spreading as a silent epidemic [10]. These ulcers not only lead to disability and a reduced quality of life for patients but also impose a significant financial burden on families and the healthcare system due to high treatment and care costs [7]. In Pakistan, multiple factors, such as limited access to healthcare, lack of awareness about diabetes management, delayed diagnosis, and inadequate treatment, contribute to the increasing prevalence of DFU [11]. Additionally, socioeconomic factors, such as poverty, low education levels, and gender inequalities, also play a significant role in exacerbating this issue [12].

Various studies investigating the prevalence of DFU have reported different findings, which has posed challenges for planning and policymaking in the prevention and treatment of this condition [13, 14, 15]. Based on this explanation, this study was conducted to estimate the overall prevalence of DFU in Pakistan.

## 2 Method

This systematic review and meta‐analysis were conducted following the Preferred Reporting Items for Systematic Reviews and Meta‐Analyses (PRISMA) guidelines [16]. The study protocol has been registered in the International Prospective Register of Systematic Reviews (PROSPERO) (CRD42025648413).

### 2.1 Search Strategy

The databases Scopus, PubMed, EMBASE, and Web of Science were systematically searched using predefined search strategies. In addition, Google Scholar and PakMediNet were searched manually to identify any potentially relevant studies not captured through database searching. The detailed search strategies for the electronic databases are provided in Table S1. In brief, the search strategy in PubMed was as follows: ((“Pakistani” [Title/Abstract] OR “Pakistan” [Title/Abstract]) AND (“epidemiology” [Title/Abstract] OR “prevalence” [Title/Abstract]) AND (“diabetic feet” [Title/Abstract] OR “DFUs” [Title/Abstract] OR “DFU” [Title/Abstract] OR “diabetic foot” [Title/Abstract] OR “diabetic foot ulcer” [Title/Abstract])).

### 2.2 Data Extraction

Two authors (ME and SS) independently extracted essential data from eligible studies and recorded them in a predesigned form. These data included the first author's name, year of publication, sample size, prevalence or frequency of DFU, study location, sampling method, study design, and inclusion and exclusion criteria. Any disagreements between the two reviewers were resolved through discussion and consensus, and if needed, by consulting a third reviewer (RGG).

### 2.3 Quality Assessment

To assess the methodological quality of the selected studies, the Joanna Briggs Institute (JBI) Critical Appraisal Tool was used. This tool includes nine items that focus on the appropriateness of the sampling frame for the target population, the sampling method used, the adequacy of the sample size, the detailed description of study participants and setting, sufficient coverage of data analysis for the identified sample, the use of valid methods to identify the condition under study, standardized and reliable measurement of the condition for all participants, appropriate statistical analysis, and the adequacy of the response rate and whether a low response rate was properly managed. Each question was answered with “Yes,” “No,” “Unclear,” or “Not applicable.” A score of 1 was assigned to “Yes” responses, whereas all other options received a score of 0. The final quality score for each study ranged from 0 to 9, with higher scores indicating better study quality [17]. The quality assessment was independently performed by two authors (ME and SS), and any disagreements between them were resolved through discussion and consensus, or by consulting a third reviewer (RGG).

### 2.4 Statistical Analysis

The collected data were analyzed using STATA version 17. To assess heterogeneity among studies, the I^2^ statistic and Cochran's Q test were used. I^2^ values were classified as low (25%), moderate (50%), and high (75%) heterogeneity. A random‐effects model was applied in cases of high heterogeneity, while a fixed‐effects model was used for low or moderate heterogeneity. Results were presented as pooled standardized scores with 95% confidence intervals and displayed in a forest plot. Publication bias was assessed visually using a funnel plot and objectively with Egger's linear regression test. If publication bias was significant, the trim‐and‐fill method was used to estimate the effect of missing studies on the overall results and determine how the corrected estimate might change. To examine whether publication year, sample size, and mean age of patients influenced the prevalence of DFU, meta‐regression analysis was performed. Additionally, sensitivity analysis was conducted to evaluate the effect of each individual study on the overall meta‐analysis results, ensuring that removing any single study did not significantly alter the final findings.

## 3 Results

**FIGURE 1:**
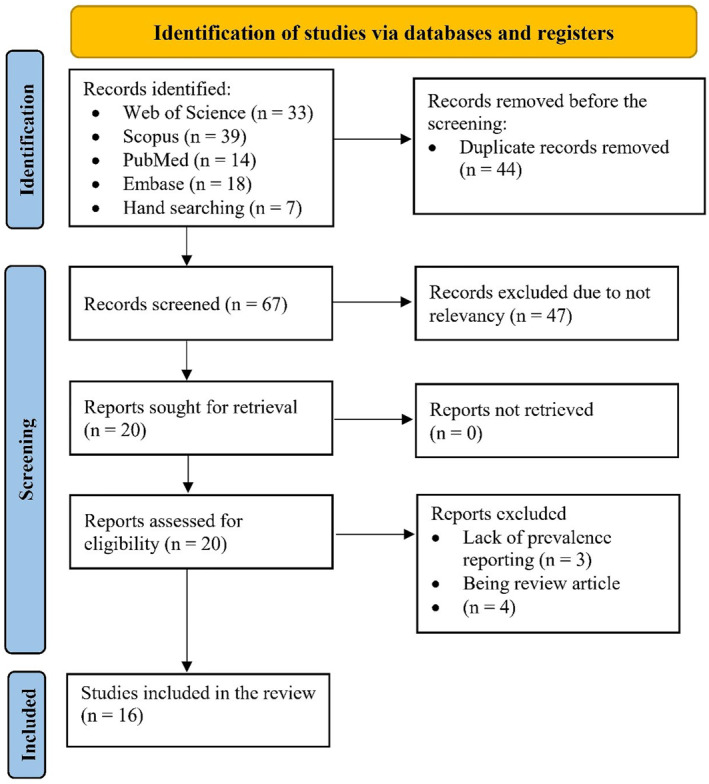
PRISMA flowchart illustrating the screening and selection process of studies.

In the initial search, 104 articles were retrieved, of which 44 were duplicates and removed. Then, the titles and abstracts of the remaining 60 articles were reviewed independently by two authors, leading to the exclusion of 51 articles due to irrelevance to the research question (47 articles), lack of precise reporting on the prevalence of DFU (3 articles), or being a review article (1 article). Additionally, 7 relevant articles were identified through manual searching. In the next step, the full texts of the remaining 16 articles were reviewed, and all 16 were included in the final analysis (Figure 1).

In this meta‐analysis, 16 studies with a total sample size of 15,333 participants were included in the analysis. All studies were cross‐sectional and published between 1999 and 2022. The sample size ranged from 151 to 2199 participants, with eight studies having a sample size of fewer than 1000 and the other eight having more than 1000 participants. Additionally, the sampling method was reported in six studies, whereas the remaining studies did not specify the sampling approach. The majority of studies were conducted in Punjab (*n* = 7) and Sindh (*n* = 4). The mean age of patients was reported in 12 studies, with an average of 52.02 years (SD = 2.44). Additionally, glycated hemoglobin (HbA1c) levels were reported in four studies, with an average of 9.2% (Table 1).

**TABLE 1:** Table of characteristics of analyzed articles.

First author (reference)	Year	Sample size			Sampling location	DFU diagnostic tool	Prevalence (%)			Place	Mean age	Sampling
		Total	Male	Female			Total	Male	Female			
Hashim [14]	1999	805	380	425	Primary care centers	Physical examination	2.1	3.42	0.94	Punjab	49.26	NA
Ahmed [18]	2004	500	—	—	Outpatient clinic	Physical examination	4	—	—	Sindh	55.2	Random sampling
Basit et [19]	2004	2199	1067	1132	University hospital	Physical examination	10.4	14.1	4.9	Sindh	51	NA
Khuwaja [20]	2004	672	—	—	Diabetes clinics	Current or past history	3.9	—	—	Sindh	52.24	NA
Hussain [21]	2010	1782	903	879	Out‐patient departments	Physical examination	3.75	5.4	2.05	Punjab	55.7	NA
Masood [22]	2013	318	—	—	Out‐patient department	Medical history	6.9	—	—	Azad Kashmir	51.83	NA
Ahmed [23]	2017	300	—	—	Government hospital	Physical examination	22.6	—	—	Sindh	NA	NA
Khan [15]	2017	230	—	—	Primary care centers	Physical examination	13.9	—	—	Pakistan	53.82	Convenience sampling
Khan [24]	2018	2052	807	1245	General hospital	Physical examination	17.3	18.2	16.8	Punjab	55	NA
Naqvi [25]	2018	151	90	61	Out‐patient department	Physical examination	37.7	58.9	55.4	NA	49.6	NA
Younis [26]	2018	1940	—	—	Diabetic clinic	Physical examination	7.0	—	—	Punjab	51.24	NA
Ejaz [13]	2020	1000	—	—	Hospital	Questionnaire	50.9	—	—	Punjab	48.26	Convenience sampling
Akhtar [11]	2022	1503	504	999	Community	Questionnaire	16.8	15.5	17.5	Punjab	51.58	Random sampling
Eijaz [27]	2022	181	81	100	Tertiary care hospital	Questionnaire	43	35.8	20	Sindh	NA	Census
Hussain [28]	2022	500	239	261	Tertiary care centers	Questionnaire	10.4	17.5	3.8	Punjab	NA	Convenience sampling
Naseer [29]	2022	1200	—	—	Hospital	Questionnaire	38	—	—	Punjab	NA	NA

The results of the methodological quality assessment showed that seven articles had moderate methodological quality, whereas the remaining articles had excellent methodological quality. Further details are provided in Table 2.

**TABLE 2:** The methodological quality of the included articles.

First author (reference)	Q1	Q2	Q3	Q4	Q5	Q6	Q7	Q8	Q9	Quality
Hashim [14]	+	U	+	+	+	+	+	+	U	Moderate
Ahmed [18]	+	+	+	+	+	+	+	+	−	Good
Basit et [19]	+	+	+	+	+	+	+	+	−	Good
Khuwaja [20]	+	+	+	+	+	+	+	+	−	Good
Hussain [21]	+	+	+	+	+	+	+	+	U	Good
Masood [22]	+	+	+	+	+	+	+	+	U	Good
Ahmed [23]	+	U	+	+	+	+	+	+	−	Moderate
Khan [15]	+	+	+	+	+	+	+	+	+	Good
Khan [24]	+	U	+	+	+	+	+	+	−	Moderate
Naqvi [25]	+	+	+	+	+	+	+	+	−	Good
Younis [26]	+	U	+	+	+	+	+	+	−	Moderate
Ejaz [13]	+	+	+	+	+	U	U	+	−	Moderate
Akhtar [11]	+	+	+	+	+	+	+	+	−	Good
Eijaz [27]	+	+	U	+	+	+	U	+	−	Moderate
Hussain [28]	+	+	+	+	+	+	+	+	U	Good
Naseer [29]	+	+	+	+	+	U	U	−	−	Moderate

*Note:* “+” = Yes, “−” = No, and “U” = Unclear.

**FIGURE 2:**
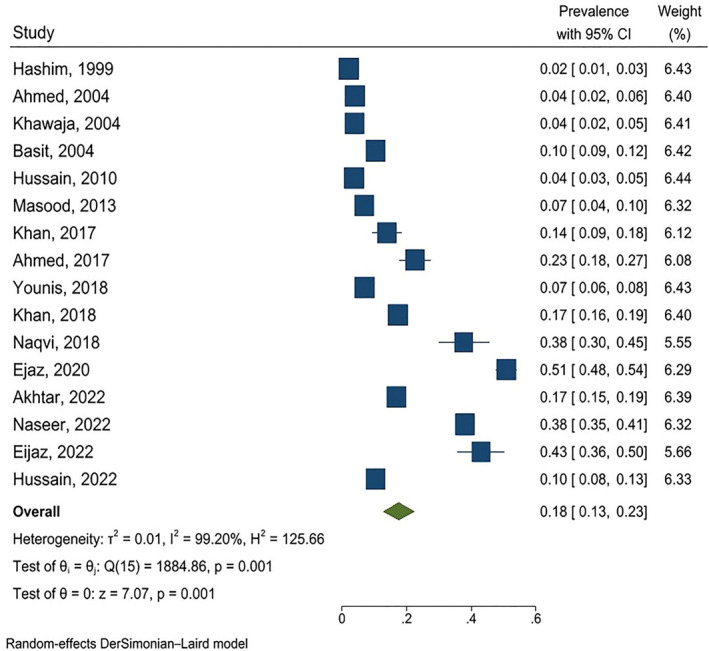
The forest plot illustrates the prevalence of DFU among patients with diabetes. Each square represents the prevalence estimate from an individual study, with the size of the squares indicating the study's weight in the overall analysis—larger squares correspond to studies with larger sample sizes. The horizontal lines depict the 95% confidence interval (95% CI) for each study's prevalence estimate. The green diamond at the bottom of the plot represents the pooled prevalence estimate derived from the meta‐analysis. The center of the diamond indicates the average prevalence, whereas its width denotes the 95% confidence interval for the overall prevalence.

**FIGURE 3:**
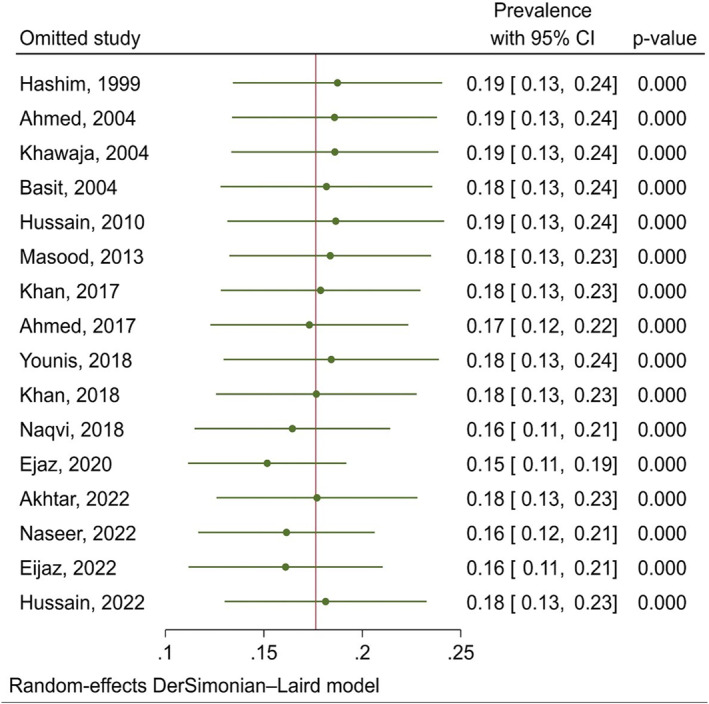
Sensitivity analysis. The green dots indicate the new prevalence estimates after omitting a study. The green horizontal lines represent the 95% confidence intervals for each prevalence estimate. The red vertical line denotes the overall pooled prevalence estimate when no study is excluded. The Prevalence with 95% CI column provides the recalculated prevalence and confidence interval after omitting each study. The *p*‐value column shows the statistical significance of the results.

The pooled prevalence of DFU was 18% (95% CI: 13–23) (I^2^ = 99.20%) (Figure 2). Sensitivity analysis showed that the results of the meta‐analysis were not influenced by any single study and demonstrated high stability (Figure 3). Subgroup analysis revealed that the prevalence was 19.4% (95% CI: 5.7–33) in Punjab and 16.6% (95% CI: 7.4–25.9) in other regions of Pakistan, with no significant difference between these locations (*p* = 0.744). Additionally, in the six studies that reported the sampling method, the prevalence of DFU was 23.1% (95% CI: 7.8–38.3) whereas in the remaining studies, it was 14.7% (95% CI: 6.4–23.1) and this difference was statistically significant (*p* = 0.034). Moreover, subgroup analysis based on study quality showed that the prevalence was 10.9% (95% CI: 7.3–14.6) in high‐quality studies and 25.7% (95% CI: 14.4–36.9) in moderate‐quality studies, and this difference was statistically significant (*p* = 0.014). In eight studies, the prevalence of DFU was reported by gender. The prevalence was 20.8% (95% CI: 8.5–33.1) in men and 14.9% (95% CI: 2.8–27) in women.

**FIGURE 4:**
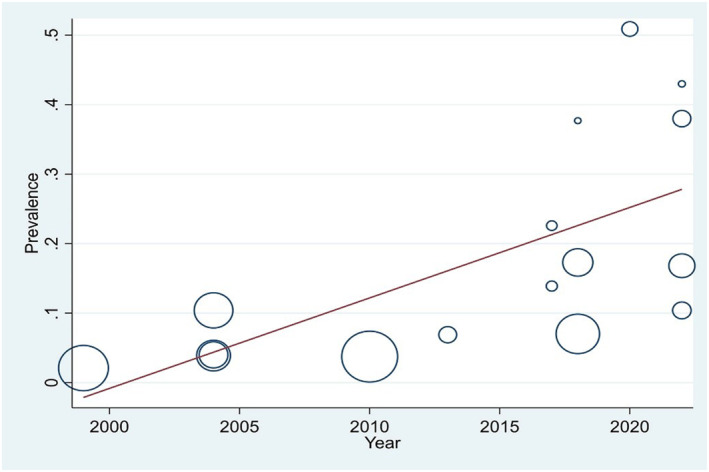
Meta‐regression of the association between the prevalence of DFU and the year of publication. The circles represent different studies. The size of the circles corresponds to the weight of each study in the meta‐regression analysis; studies with larger sample sizes have larger circles. The red line is a regression line, indicating the overall trend between the year of publication and the prevalence of DFU.

Meta‐regression was performed to assess the association between the pooled prevalence of DFU and variables such as the year of publication, mean patient age, and study sample size. The results indicated that only the year of publication was significantly associated with prevalence. Specifically, the prevalence of DFU showed a significant increase from 1999 to 2022 (*p* = 0.007) (Figure 4).

**FIGURE 5:**
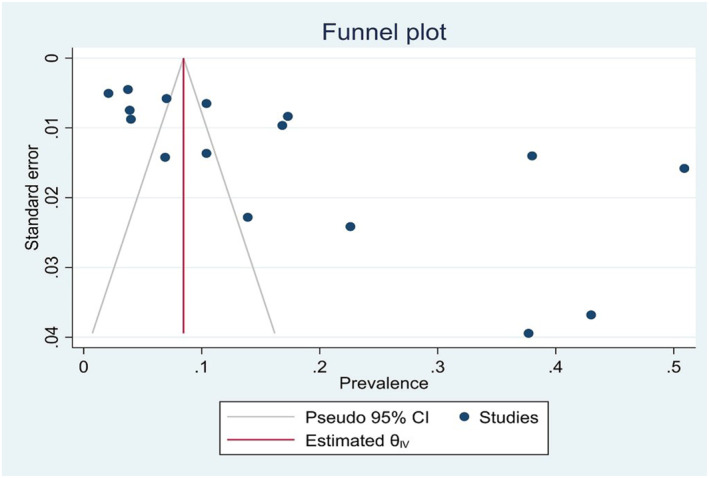
Publication bias. The *X*‐axis represents the prevalence. The *Y*‐axis shows the standard error, which decreases as the sample size increases. The blue dots represent different studies. The red line indicates the estimated overall effect. The two gray diagonal lines represent the pseudo 95% confidence interval.

To assess publication bias, a funnel plot and Egger's test were used. The results showed that Egger's test was statistically significant, and the funnel plot indicated an asymmetrical distribution of studies, suggesting potential publication bias. To address this issue, the trim‐and‐fill method was applied. After imputing one missing study, the estimated prevalence changed slightly from 0.179 to 0.189. Given this minor change, the impact of publication bias on the overall prevalence estimate was considered negligible (Figure 5).

## 4 Discussion

The findings of this meta‐analysis indicate that the overall prevalence of DFUs among diabetic patients in Pakistan is 18% (95% CI: 13–23). In this meta‐analysis, 16 studies with a total of 15,333 participants were examined. All studies were cross‐sectional and were published between 1999 and 2022. The mean age of the patients was 52.02 years with a standard deviation of 2.44, and the mean glycated hemoglobin (HbA1c) level was reported in four studies, averaging 9.2%. These findings suggest that DFU are a common issue among diabetic patients in Pakistan. Other meta‐analysis studies in Pakistan have also reported a high prevalence of DFU. For example, a meta‐analysis conducted by Akhtar et al. (2020) reported a prevalence of 12% in Pakistan, which is close to the findings of this study [12]. These findings are also in line with previous studies that have reported the prevalence of DFU in low‐ and middle‐income countries (LMICs) [2, 30, 31].

In this study, a very high level of heterogeneity (I^2^ = 99%) was observed among the included studies, indicating substantial differences between them. This heterogeneity may be attributed to variations in study design, sample size, geographic regions, and patient characteristics as well as the diagnostic criteria used to identify DFUs. In addition, differences in sampling methods and reporting practices may have contributed to this variability. It is noteworthy that such high levels of heterogeneity are very common in prevalence meta‐analyses as primary studies often vary considerably in terms of population, setting, and methodology. Therefore, this high heterogeneity should be taken into account when interpreting the pooled prevalence estimate.

Globally, the prevalence of DFU has been reported to be significantly lower. A study conducted by Zhang et al. (2020) estimated the global prevalence of DFU to be around 6.3%, with the highest prevalence in North America (13%) and the lowest in Oceania (3%) [31]. Similarly, the meta‐analysis by Sahu et al. in India reported an overall prevalence of 6.2%, with a significantly higher prevalence in hospital‐based studies (7.5%) compared to community‐based studies (2.5%) [32]. These differences may be attributed to variations in healthcare systems, access to medical care, patient awareness of diabetes and its complications, socioeconomic factors, limited access to healthcare services, and the high prevalence of uncontrolled diabetes in these regions.

The results of this meta‐analysis also revealed that the prevalence of DFU varies across different provinces of Pakistan. Specifically, the prevalence in Punjab (19.4%) was slightly higher than in other regions (16.6%), although this difference was not statistically significant. However, it is close to the overall prevalence of DFU in Punjab reported by Akhtar, which was 16.83% (95% CI: 14.9%–18.7%) [11]. This finding may be attributed to differences in access to healthcare, patient awareness levels, and the prevalence of diabetes‐related risk factors across regions. Previous studies have also indicated that geographical and regional factors can significantly influence the prevalence of diabetes complications [14, 33]. The findings showed that the prevalence of DFU was higher in men than in women. This difference may be due to the higher rates of smoking and peripheral arterial disease in men [34, 35]. Additionally, men with diabetes are often less likely to seek medical care in the early stages, leading to delayed diagnosis and an increased risk of developing severe foot ulcers [36].

One of the noteworthy findings of this study was the significant difference in the prevalence of DFU based on the reporting of the sampling method. In studies that reported their sampling method, the prevalence of DFU was significantly higher (23.1%) compared to those that did not report their sampling method (14.7%). This difference may indicate that studies with more precise sampling methods were more likely to identify patients with more severe conditions or complications. This finding underscores the importance of accurately reporting methodology in future studies to enable more precise interpretation and comparison of results. Based on the results of meta‐regression, the prevalence of DFU has shown a significant upward trend in Pakistan from 2000 to 2022. It should be noted that this meta‐regression was based on aggregated study‐level data. Therefore, a causal relationship cannot be fully inferred, and the observed increase in DFU prevalence over time may partly reflect improvements in case detection, reporting accuracy, or changes in study designs rather than a true rise in disease occurrence. Furthermore, the subgroup analysis showed that the prevalence of DFU was significantly lower in high‐quality studies (10.9%) compared to moderate‐quality studies (25.7%). This suggests that lower study quality may have led to overestimation of prevalence due to methodological biases. Therefore, findings from lower‐quality studies should be interpreted with caution, and future research should adopt more rigorous methodologies to provide more reliable estimates.

The high prevalence of DFU in Pakistan arises from several interconnected factors. Pakistan has one of the highest global burdens of diabetes, with the 2025 International Diabetes Federation report estimating that almost one‐third of adults are affected [37]. Glycemic control remains suboptimal in most patients [38, 39], which increases the risk of complications such as neuropathy, vascular disease, and delayed wound healing [24]. Limited awareness of foot care, delays in recognizing early symptoms, and limited availability of specialized services further contribute to late presentation and poor outcomes [40, 41]. These challenges are compounded by financial hardship and low literacy, which restricts opportunities for effective self‐management [42]. Together, these factors explain the disproportionately high prevalence of DFU in Pakistan compared to global averages.

## 5 Strengths and Limitations

Strengths: This study has several notable strengths. For the first time, the results of multiple studies on diabetic foot ulcer (DFU) prevalence in Pakistan have been pooled and reported together. To the best of our knowledge, no previous study has conducted a meta‐analysis on this topic, making our work the most comprehensive synthesis of available evidence to date.

This study had several limitations: (1) There was very high heterogeneity (I^2^ = 99%) among the included studies. Although this is common in prevalence meta‐analyses, it may affect the precision of the pooled estimate and should be considered when interpreting the results. (2) Some studies did not use precise sampling methods or provide sufficient methodological details, which may impact the reliability of the findings. (3) The study exhibited publication bias, although the trim‐and‐fill method indicated that its effect was negligible. (4) Most studies were conducted in Punjab and Sindh, with limited data available for other regions of Pakistan, particularly rural areas. (5) There is a potential risk of misclassification of DFU cases in the included studies as diagnostic criteria and reporting practices may have varied across studies. (6) Important individual‐level factors, such as diabetes duration and glycemic control, were not consistently reported across studies; therefore, we were unable to adjust for these potential confounders, which may have influenced the observed prevalence estimates.

## 6 Conclusion

In this meta‐analysis, the overall prevalence of DFU in Pakistan was 18%, showing a significant upward trend from 2000 to 2022. The findings revealed regional, gender‐based, and methodological differences in prevalence, highlighting the importance of improving screening methods and reporting accuracy. The results of this study emphasize the need for targeted prevention strategies and improved management of DFUs in Pakistan.

## Author Contributions

**Reza Ghanei Gheshlagh:** conceptualization, methodology, formal analysis, data curation, writing – original draft, writing – review and editing. **Simin Sharafi:** conceptualization, supervision, investigation, validation, writing – original draft, writing – review and editing. **Mohammad Esmaeelzadeh:** methodology, investigation, data curation, writing – original draft, writing – review and editing. **Kainat Asmat:** supervision, validation, writing – review and editing.

## Funding

The authors have nothing to report.

## Ethics Statement

The authors have nothing to report.

## Consent

The authors have nothing to report.

## Conflicts of Interest

The authors declare no conflicts of interest.

## Supporting information


**Table S1:** Database search strategies and number of retrieved records.

## Data Availability

All data on diagnostic yield analyzed during the current study are available in the main text or supplementary material.
