# Supplementary material for: Diabetic Foot Ulcers in Pakistan: A Silent Epidemic?—A Meta‐Analysis of Observational Studies
Source: J Foot Ankle Res. 2025 Dec 25;18(4):e70117. doi: 10.1002/jfa2.70117 (PMC12740441; doi:10.1002/jfa2.70117)
Supplement: Supplementary file 1 — Table S1: Database search strategies and number of retrieved records. [file JFA2-18-e70117-s001.docx]

**Supplementary Table 1.** Database search strategies and number of retrieved records

| **Scopus (n=39)** | ( TITLE-ABS-KEY ( pakistani ) OR TITLE-ABS-KEY ( pakistan ) ) AND (TITLE-ABS-KEY ( epidemiology ) OR TITLE-ABS-KEY ( prevalence ) ) AND (TITLE-ABS-KEY ( diabetic AND feet ) OR TITLE-ABS-KEY ( dfus ) OR TITLE-ABS-KEY ( dfu ) OR TITLE-ABS-KEY ( diabetic AND foot ) OR TITLE-ABS-KEY ( diabetic AND foot AND ulcer ) ) |
| --- | --- |
| **PubMed (n=14)** | (("Pakistani"[Title/Abstract] OR "Pakistan"[Title/Abstract]) AND ("epidemiology"[Title/Abstract] OR "prevalence"[Title/Abstract]) AND ("diabetic feet"[Title/Abstract] OR "DFUs"[Title/Abstract] OR "DFU"[Title/Abstract] OR "diabetic foot"[Title/Abstract] OR "diabetic foot ulcer"[Title/Abstract])) |
| **WOS (N = 33)** | # Searches:  1: TS=(Pakistani) Date Run: Wed Feb 05 2025 22:05:34 GMT+0330 (Iran Standard Time) Results: 15654  2: TS=(Pakistan) Date Run: Wed Feb 05 2025 22:05:54 GMT+0330 (Iran Standard Time) Results: 85229  3: TS=(epidemiology) Date Run: Wed Feb 05 2025 22:06:06 GMT+0330 (Iran Standard Time) Results: 508920  4: TS=(prevalence) Date Run: Wed Feb 05 2025 22:06:18 GMT+0330 (Iran Standard Time) Results: 1369957  5: TS=(diabetic feet) Date Run: Wed Feb 05 2025 22:06:35 GMT+0330 (Iran Standard Time) Results: 23440  6: TS=(DFUs) Date Run: Wed Feb 05 2025 22:06:47 GMT+0330 (Iran Standard Time) Results: 1636  7: TS=(DFU) Date Run: Wed Feb 05 2025 22:06:57 GMT+0330 (Iran Standard Time) Results: 2656  8: TS=(diabetic foot) Date Run: Wed Feb 05 2025 22:07:07 GMT+0330 (Iran Standard Time) Results: 23443  9: TS=(diabetic foot ulcer) Date Run: Wed Feb 05 2025 22:07:17 GMT+0330 (Iran Standard Time) Results: 13720  10: #9 OR #8 OR #7 OR #6 OR #5 Date Run: Wed Feb 05 2025 22:07:54 GMT+0330 (Iran Standard Time) Results: 23770  11: #3 OR #4 Date Run: Wed Feb 05 2025 22:08:01 GMT+0330 (Iran Standard Time) Results: 1748707  12: #1 OR #2 Date Run: Wed Feb 05 2025 22:08:06 GMT+0330 (Iran Standard Time) Results: 93171  13: #12 AND #11 AND #10 Date Run: Wed Feb 05 2025 22:08:12 GMT+0330 (Iran Standard Time) |
| **Embase (N = 18)** | ('pakistan' OR 'pakistani').ti,ab,kw.  AND  ('epidemiology' OR 'prevalence').ti,ab,kw.  AND  (('diabetic foot*' OR 'diabetic foot ulcer*' OR 'DFU' OR 'DFUs').ti,ab,kw.) |
